# Self-compatibility in ‘Zaohong’ Japanese apricot is associated with the loss of function of pollen *S* genes

**DOI:** 10.1007/s11033-013-2765-2

**Published:** 2013-09-24

**Authors:** Pei-Pei Wang, Zhi-Hong Gao, Zhao-Jun Ni, Zhen Zhang, Bin-Hua Cai

**Affiliations:** College of Horticulture, Nanjing Agricultural University, No.1 Weigang, Nanjing City, 210095 Jiangsu Province People’s Republic of China

**Keywords:** Japanese apricot, Self-compatibility (compatible) (SC), Self-incompatibility (incompatible) (SI), *S*-*RNase*, *SFB*, *PmF*-*box*

## Abstract

While most Japanese apricot (*Prunus mume* Sieb. et Zucc.) cultivars display typical *S*-*RNase*-based gametophytic self-incompatibility, some self-compatible (SC) cultivars have also been identified. In this study, we confirmed SC of ‘Zaohong’ through replicated self-pollination tests. Cross-pollination tests showed that SC of ‘Zaohong’ was caused by a loss of pollen function, so we determined that the *S*-genotype of ‘Zaohong’ was *S*_*2*_ *S*_*15*_. Sequence analysis of the *S*-haplotypes of ‘Zaohong’ showed no mutations which were likely to alter gene function. Furthermore, expression analysis based on RT-PCR of *S*-locus genes revealed no differences at the transcript level when compared with ‘Xiyeqing’, a self-incompatible cultivar with the same *S* haplotypes. In addition, except for *S*-locus genes, a new type of F-box gene encoding a previously uncharacterised protein with high sequence similarity (61.03–64.65 %) to *Prunus SFB* genes was identified. Putative structural regions of *PmF*-*bo*x genes have been described, corresponding to regions in *PmSFB* alleles, but with some sequence variations. These results suggest that SC in ‘Zaohong’ occurs in pollen, and that other factors outside the *S*-locus, including *PmF*-*box* genes, might be associated with the loss of function of pollen *S* genes.

## Introduction

The *S*-*ribonuclease* (*S*-*RNase*)-based gametophytic self-incompatibility (GSI) found in the Solanaceae [1, 2], Scrophulariaceae [3] and Rosaceae [4, 5], is a genetic mechanism in flowering plants that prevents inbreeding, promotes out-crossing, and is often controlled by the highly polymorphic, multi-allelic *S*-locus [6]. In these three families, the *S*-locus is comprised of the pistil *S*-*RNase* [2, 3] and the pollen-expressed *SFB/SLF* (*S*-haplotype-specific F-box/*S*-locus F-box) genes [7–10]. Different variants of the *S*-locus are thus described as ‘haplotypes’. Pollen inhibition occurs when the same *S*-haplotype is expressed in both pollen and pistil [6].

In the Solanaceae, Scrophulariaceae, and Rosaceae, active *S*-*RNases* expressed in the style are essential for the inhibition of pollen-tube growth during the incompatibility response and are thought to act via the degradation of ribosomal RNA within the pollen tube [11]. F-box proteins meanwhile, are components of the SCF complex, which regulates protein degradation via the ubiquitin/26S proteasome pathway [7–15]. Therefore, the identification of *S*-linked F-box genes expressed in pollen raises the possibility that the ubiquitin/26S proteasome pathway plays a key role in self/non-self pollen discrimination in *S*-*RNase*-based GSI. The type of interaction between *S*-*RNase* and *SFB* might determine SI or SC [16].

In most *Prunus* fruit trees, natural or artificially-produced self-compatible (SC) mutants have been used to study the molecular basis of the *S*-*RNase*-based GSI system, which is attributed to the loss of function of the factors at the *S* locus [17] or external to the *S* locus [6]. Stylar-part mutations (SPMs) at the *S*-locus have been discovered in several SC species such as peach (*Prunus persica*) [17], almond (*Prunus dulcis*) [18], sour cherry (*Prunus cerasus*) [19] and Japanese pear (*Pyrus serotina*) [5, 20], whereas pollen-part mutations (PPMs) have also been discovered in peach (*P*. *persica*) [17], sour cherry (*P*. *cerasus*) [19], as well as in sweet cherry (*Prunus avium*) [12, 21, 22] and in Japanese apricot (*Prunus mume*) 12, 23]. SPMs and PPMs in SC mutants have mainly been found to be associated with deletions or insertions in *S*-*RNase* and *SFB* genes, respectively. Finally, SC has also been associated with mutations at a modifier locus that is unlinked to the *S*-locus in various *Prunus* species [24–28].

The Japanese apricot, which originated in southeast China, belongs to the Rosaceae family and is an important economical and deciduous fruit crop that exhibits GSI [29–31]. The fruit of Japanese apricot has always been a valuable processing material used in the food and beverage industries [32]. Since Japanese apricot exhibits GSI, pollinator cultivars are required in commercial production to ensure fruit set. However, different to other *Prunus* species, the Japanese apricot blooms very early in the spring, so that low temperature, wind conditions and available insects will restrict pollination. Thus, SC cultivars have a horticultural advantage over SI cultivars because no cross-pollinatorer is required, especially for the Japanese apricot [30, 31]. Therefore, it is necessary to search for different mechanisms of SC in order to avoid inbreeding depression.

In this study, we used data derived from self-pollination tests to confirm that ‘Zaohong’ showed SC. The objective was further to investigate the cause of this SC in ‘Zaohong’ through cross-pollination tests, and to analyse both *S*-*RNase* and *SFB* genes and other factors outside the *S*-locus.

## Materials and methods

### Plant material

‘Zaohong’ (*S*_*2*_ *S*_*15*_, SC) [32] and Xiyeqing (*S*_*2*_ *S*_*15*_, SI) [32] trees grown in the National Field Genebank for Japanese apricot cultivars located at Nanjing Agricultural University were used in this study. Young leaves, pollen grains, and styles with stigmas were collected in April 2012, frozen in liquid nitrogen, and stored at −70 °C until use.

### Field pollination tests

Self-pollination and cross-pollination tests of ‘Zaohong’ and ‘Xiyeqing’ were carried out in the field. Pollen samples were collected from flowers at the balloon stage and dried at room temperature, then stored in a desiccator at 4 °C until use. Before pollination, pollen viability was examined and the stamens were removed by forceps. Flower buds were emasculated before anthesis and covered with paper bags to avoid contamination. The number of fruits set was recorded 60 days after pollination, and fruit rate was calculated using data collected over a 3-year survey. In terms of the standard, fruit set ≥5 % was considered to be SC, whereas that <5 % was considered to be SI [31].

### DNA and RNA extraction

Total genomic DNA was extracted from frozen young leaves of ‘Zaohong’ using the cetyl trimethyl ammonium bromide (CTAB) method according to Wang et al. [33]. Total RNA was extracted from pollen grains, styles and leaves of ‘Zaohong’ and ‘Xiyeqing’ as described in Tao et al. [34].

### PCR of *S*-*RNase* alleles

For the identification of the *S*-genotype of ‘Zaohong’, PCR amplification was carried out using a *Prunus* *S*-*RNase* consensus primer pair, Pru-C2 [34] and PCE-R [35], designed from the second and third conserved regions of *Prunus S*-*RNase* genes, respectively (Table 1). To analyse further the structure of *S*-*RNase* genes, *S*_*2*_-*RNase* and *S*_*15*_-*RNase* were amplified using the *Prunus S*-*RNase* consensus primer pair, SRc-F [36] and PM-C5 [37], designed from the signal peptide and the fifth conserved region of *Prunus S*-*RNase* genes, respectively (Table 1). PCR reaction mixtures and conditions were identical to those described by Xu et al. [38]. PCR was performed in a 25 μL reaction volume containing 69 ng genomic DNA, 2.0 μL 10 × PCR buffer (TaKaRa, Kyoto, Japan), 1.5 mM MgCl_2_, 0.15 mM dNTPs, 0.1 μM each primer and 1 U Taq DNA polymerase (TaKaRa, Japan) in a PTC-100 thermal cycler (MJ Research, Cambridge, MA, USA). PCR reactions were run with a programme of 35 cycles at 94 °C for 30 s, 60 °C for 40 s and 72 °C for 90 s, with an initial denaturation at 94 °C for 3 min and a final extension of 72 °C for 10 min. The PCR products were separated by 1.2 % agarose gel electrophoresis in 1 × TAE buffer and observed using an ultraviolet light system (FR-200, Peiqing, China).

**Table 1:** Sequences of primers used in this study

Primer	Sequence (from 5′ to 3′)	Notes
ActF1	ATGGTGAGGATATTCAACCC	[9]
ActR1	CTTCCTGTGGACAATGGATGG	[9]
Pru-C2	CTATGGCCAAGTAATTATTCAAACC	[34]
PCE-R	TGTTTGTTCCATTCGCYTTCCC	[35]
F-BOX5′A	TTKSCHATTRYCAACCKCAAAAG	[39]
SFB-C1F	RTTCGRTTTCTDTTTACRTG	[35]
SRc-F	CTCGCTTTCCTTGTTCTTGC	[36]
PM-C5	CATAATACCACTTCATGTAA	[37]
Pm-Vb	ATCCAAGCAAGTTCTTGAAACA	This study
SFB2A	GTTCGGTTTCTATTTACGTG	This study
SFB2B	ATGGGTCGAAGAGATTTAGC	This study
SFB15A	ATGCAAGTCGTGGAGTGATT	This study
SFB15B	CGATAAGGGCGTAGCAGATC	This study
PmF1	AAGGATAAAGAAAAGGACGC	This study
PmF2	CTACGAAAACTACGAAGACT	This study

### PCR of *SFB* alleles

PCR amplification of *SFB* alleles in ‘Zaohong’ was performed using the primers, F-BOX5′A [39], SFB-C1F [40] and Pm-Vb, which were designed from conserved regions upstream from the intron, the F-box motif and upstream from HVb of *Prunus SFB* genes, respectively (Table 1). PCRs were performed in a 20 μL reaction volume containing 70 ng genomic DNA, 2.0 μL 10 × PCR buffer (TaKaRa, Kyoto, Japan), 1.5 mM MgCl_2_, 0.2 mM dNTPs, 0.1 μM of each primer and 1 U Taq DNA polymerase (TaKaRa, Japan). PCR reactions were run with a programme of 35 cycles at 94 °C for 1 min, 56 °C for 1 min and 72 °C for 90 s, with an initial denaturing at 94 °C for 3 min and a final extension of 72 °C for 10 min. The PCR products were separated by 1.2 % agarose gel electrophoresis in 1 × TAE buffer and observed using an ultraviolet light system (FR-200, Peiqing, China).

### RT-PCR

One microgram of total RNA from pollen grains, styles, and leaves was used for first-strand cDNA synthesis using a PrimeScript RT reagent Kit with gDNA Eraser (TaKaRa), with the resulting cDNAs acting as templates for RT-PCR. RT-PCRs of the *S*-*RNase*, *SFB* and *Actin* genes were performed using the primer pairs Pru-C2/PCE-R, SFB-C1F/Pm-Vb and ActF1/ActR1, respectively (Table 1). In addition, the specific primer pair SFB2A and SFB2B was used for the amplification of *PmSFB*_*2*_, and SFB15A and SFB15B for the amplification of *PmSFB*_*15*_. For the newly-described *PmF*-*box* genes, specific primers PmF1 and PmF2 were designed from conserved regions of these genes. PCR reaction mixtures and conditions used were as described for genomic DNA amplification.

### Cloning and sequencing of genomic PCR products and cDNAs

All PCR products and cDNA fragments were excised from 1.2 % agarose gels and purified using the Agarose Gel DNA Purification Kit (TaKaRa, Japan). The purified products were cloned into the PMD19-T vector (TaKaRa) following the manufacturer’s instructions and transformed into *Escherichia coli* DH5α. Target clones were sequenced on an ABI Prism 3700 DNA analyser (ABI, 3730, USA) using the M13 primer and the Big Dye Terminator Version 3.1. To obtain accurate sequences and avoid PCR amplification errors, four positive clones of each fragment were sequenced by the Invitrogen Company (Shanghai, China).

### Sequence and phylogenetic analysis

Homology searches were performed using BLAST (National Center for Biotechnology Information). Sequence alignments were performed using DNAMAN (version 5.2; Lynnon Biosoft). A phylogenetic tree was then generated using MEGA version 3.1 using P-distance and neighbour-joining methods with 1,000 bootstrap replication tests.

## Results

### Pollination tests

To confirm the SC/SI of ‘Zaohong’ and ‘Xiyeqing’, self-pollination tests were carried out (Table 2). For ‘Zaohong’ and ‘Xiyeqing’, the number of fruits set from 210 flower buds were 34 and 0, respectively. According to the criteria that cultivars with a fruit set ≥5 % showed SC, the fruit set of 16.19 % indicated that ‘Zaohong’ showed SC. Apparently ‘Xiyeqing’ with a fruit set of 0 % showed SI.

**Table 2:** Rates of fruit setting in self- or cross-pollination of ‘Zaohong’ and ‘Xiyeqing’

♀	♂	Number of flowers pollinated	Number of fruit set	Rate of fruit set (%)
Zaohong (*S*_*2*_ *S*_*15*_)	Zaohong (*S*_*2*_ *S*_*15*_)	210	34	16.19
Xiyeqing (*S*_*2*_ *S*_*15*_)	Xiyeqing (*S*_*2*_ *S*_*15*_)	210	0	0
Zaohong (*S*_*2*_ *S*_*15*_)	Xiyeqing (*S*_*2*_ *S*_*15*_)	210	0	0
Xiyeqing (*S*_*2*_ *S*_*15*_)	Zaohong (*S*_*2*_ *S*_*15*_)	210	26	12.38

In addition, ‘Zaohong’ was reciprocally crossed with ‘Xiyeqing’, a cultivar with the same *S* haplotypes (*S*_*2*_ *S*_*15*_). As presented in Table 2, no fruits were obtained when ‘Zaohong’ was used as a hybrid female parent. However, the number of fruits set was 26 when ‘Zaohong’ was used as a hybrid male parent. Therefore, ‘Zaohong’ × ‘Xiyeqing’ and ‘Xiyeqing’ × ‘Zaohong’ possessing different fruit sets were 0 and 12.38 %, respectively, i.e., ‘Zaohong’ × ‘Xiyeqing’ displayed cross-incompatibility, but ‘Xiyeqing’ × ‘Zaohong’ showed cross-compatibility.

### *S*-*RNase* and *SFB* alleles from ‘Zaohong’

**Fig. 1:**
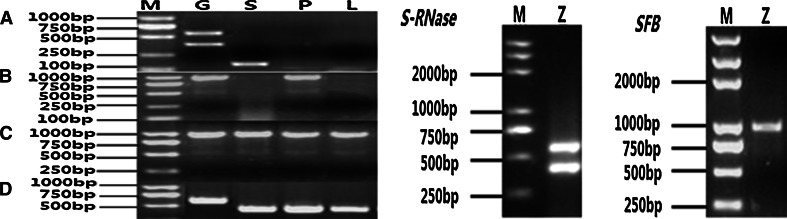
PCR amplification of *S*-*RNase* and *SFB* alleles from ‘Zaohong’ and expression analysis for *PmS*-*RNase* (*a*), *PmSFB* (*b*), *PmF*-*box* genes (*c*) and *Actin* genes (*d*) in pollen (*P*), styles (*S*), and leaves (*L*). *M* is DNA marker, *Z* is Zaohong and *G* is genomic DNA. *a* RT-PCR of *S*-*RNase* alleles. *b* RT-PCR of *SFB* alleles. *c* RT-PCR of *PmF*-*box* alleles. *d* RT-PCR of *Actin* genes

Two DNA bands were obtained from ‘Zaohong’ using the primer pair Pru-C2 and PCE-R (Fig. 1, *S*-*RNase*). Further cloning and sequencing confirmed that the *S*-genotype of ‘Zaohong’ was *S*_*2*_ *S*_*15*_, the same as the identification by Xu et al. [32].

PCR amplification of the *SFB* alleles was performed using the primer pair SFB-C1F and Pm-Vb, designed from conserved regions in the F-box motif and upstream of HVb of *Prunus SFB* genes, respectively. Only one amplified fragment of approximately 1,000 bp was obtained from ‘Zaohong’ genomic DNA (Fig. 1 *SFB*). However, sequencing results showed that the band contained two different sequences, indicating the presence of two *SFB* alleles in ‘Zaohong’. Sequence analysis and BLAST searches in GenBank confirmed that two *PmSFB* genes, *PmSFB*_*2*_ (GenBank accession number: JQ356589) and *PmSFB*_*15*_, were identified in ‘Zaohong’.

### Specific expression of *S*-*RNase* and *SFB* genes

**Fig. 2:**
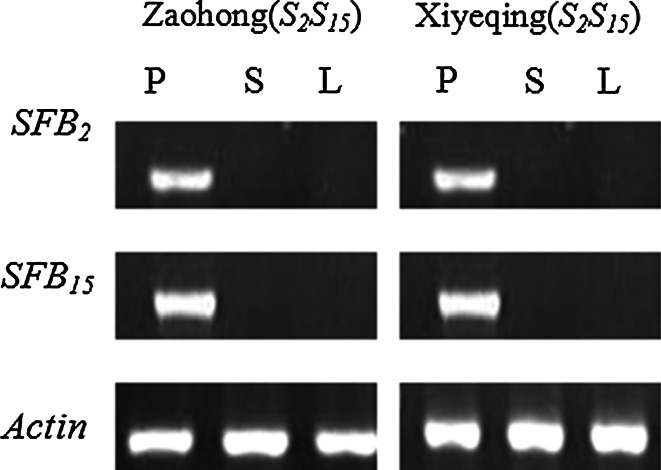
RT-PCR analysis of the *SFB*_*2*_, *SFB*_*15*_ and *Actin* genes in pollen (*P*), styles (*S*) and leaves (*L*) of ‘Zaohong’ (*S*_*2*_ *S*_*15*_) and ‘Xiyeqing’ (*S*_*2*_ *S*_*15*_)

RT-PCR of *S*-*RNase*, *SFB* and *Actin* genes was performed using total RNA from pollen grains, styles and leaves of ‘Zaohong’ with the primers Pru-C2/PCE-R, SFB-C1F/Pm-Vb and ActF1/ActR1, respectively (Fig. 1). RT-PCR analysis of *Actin* genes amplified fragments of the same size, and which were shorter than the fragments amplified from genomic DNA, due to the absence of introns (Fig. 1d). The results, therefore, confirmed that the RNA preparations were free from genomic DNA contamination. For RT-PCR of *S*-*RNase* alleles, amplified fragments were only found in stylar RNA and genomic DNA (Fig. 1a). Furthermore, amplified fragments from the style were shorter those from genomic DNA due to the absence of the second intron. RT-PCR of *SFB* alleles from pollen grain RNA however, produced a fragment of the same size as that of genomic DNA, but no amplification was seen with leaf RNA or stylar RNA samples (Fig. 1b). Together, these results indicate that the *S*-*RNase* and *SFB* alleles identified in ‘Zaohong’ are specifically expressed in style and pollen, respectively (Fig. 2).

### Sequence analysis of *S*-*RNase* and *SFB* genes

**Fig. 3:**
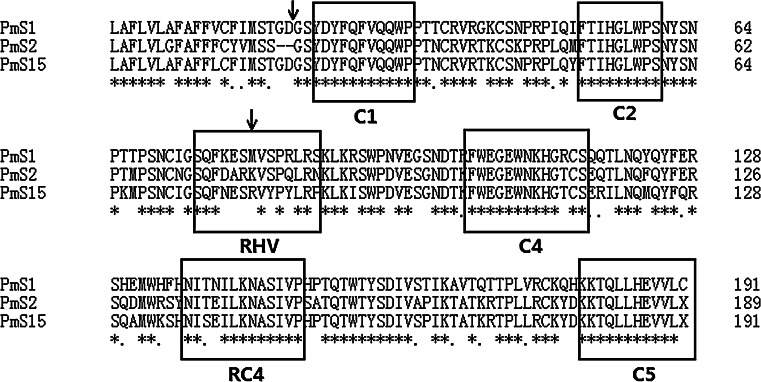
Alignment of the predicted amino acid sequences of *PmS*-*RNase* alleles. *Asterisks*, *dots* and *dashes* indicate conserved amino acid residues, conservative substitutions and gaps, respectively. Conserved (*C1*, *C2*, *C3*, *RC4* and *C5*) and hypervariable (*RHV*) regions are *boxed*, and *arrows* indicate the position of the introns. GenBank accession numbers: *PmS*_*1*_ (AB364462). *PmS*_*2*_ and *PmS*_*15*_ were determined in this study

Using the primer pair, Pru-C2 and PCE-R, we confirmed that the *S*-genotype of ‘Zaohong’ was *S*_*2*_ *S*_*15*_ based on sequence analysis. To analyse further the structure of *S*-*RNase* alleles, we cloned PCR products of almost full-length sequences of *S*-*RNase* genes that contained the first and second introns, using SRc-F and PM-C5. These primers were designed from the signal peptide and the fifth conserved region of *Prunus S*-*RNase* genes, respectively. *S*_*2*_-*RNase* and *S*_*15*_-*RNase* were successfully cloned using SRc-F and PM-C5. Compared with the predicted amino acid sequence of *PmS*_*1*_-*RNase*, the putative *S*_*2*_-*RNase* and *S*_*15*_-*RNase* genes showed the typical features of *Prunus S*-*RNase* genes with five conserved domains (C1, C2, C3, RC4 and C5) and one RHV (Fig. 3). These results indicated that there were no mutations or insertions in *S*_*2*_-*RNase* and *S*_*15*_-*RNase* which could disrupt the typical features.

**Fig. 4:**
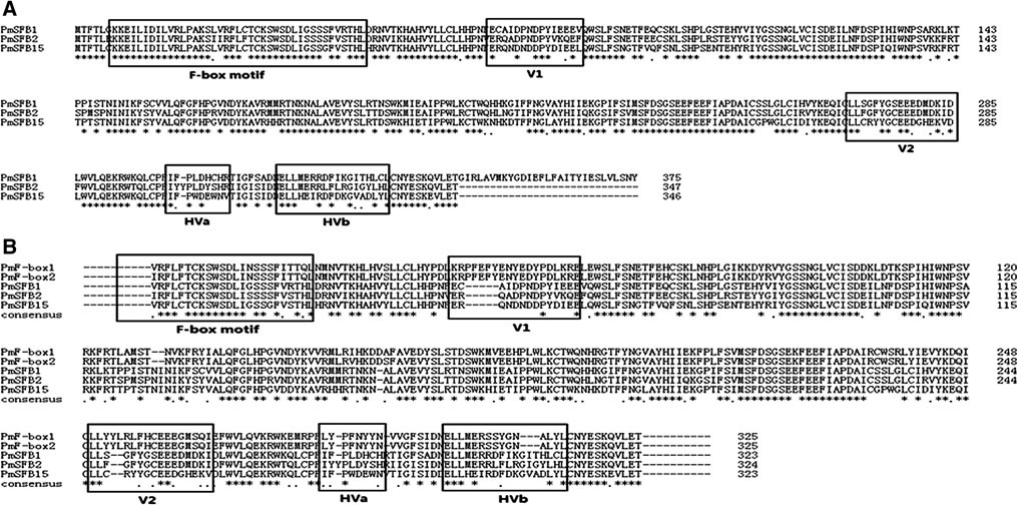
**a** Alignment of the predicted amino acid sequences of *PmSFB* alleles. **b** Alignment of the predicted amino acid sequences of *PmF*-*box* genes, *PmSFB*_*1*_, *PmSFB*_*2*_ and *PmSFB*_*15*_. F-box motif, variable (*V1* and *V2*) and hypervariable (*HVa* and *HVb*) regions are *boxed*. GenBank accession numbers: *PmSFB*_*1*_ (AB101440). *PmSFB*_*2*_ and *PmSFB*_*15*_ were determined in this study. *Asterisks*, *dots* and *dashes* indicate conserved amino acid residues, conservative substitutions and gaps, respectively

To analyse the structure of the *PmSFB* genes, PCRs were performed using F-BOX5′A and Pm-Vb. As with *PmSFB*_*1*_, *PmSFB*_*2*_ and *PmSFB*_*15*_ of ‘Zaohong’ were found to have an F-box motif and four (hyper-) variable regions (V1, V2, HVa and HVb) (Fig. 4a). Because the corss-pollination tests indicated that SC in ‘Zaohong’ occurred in pollen, the specific expression of *SFB* genes was tested using RT-PCR. Figure 2 showed that *SFB* genes of ‘Zaohong’ have the same expression levels in pollen as *SFB* genes of ‘Xiyeqing’. Taken together, these findings indicate that neither the coding sequence nor the transcriptional level is altered in *SFB* genes of ‘Zaohong’.

### Sequence analysis of *PmF*-*box* genes

*PmF*-*box1* (GenBank accession number: JX141276) and *PmF*-*box2* (GenBank accession number: JX141277), which are 98.46 % identical at the amino acid level, were identified from ‘Zaohong’ using SFB-C1F and Pm-Vb primers. The amino acid identities between *PmSFB* and the *PmF*-*box* genes ranged from 61.03 to 64.65 % (not shown). Interestingly, BLAST searches in GenBank showed that the amino acid sequences of *PmF*-*box* genes have a high similarity to *ParF*-*box2* (95.69 %). Similar to the structure of *PmSFB* genes, both *PmF*-*box* genes contain a putative F-box motif, two variable regions (V1 and V2) and two hypervariable regions (HVa and HVb) (Fig. 4b). Despite this, there were several notable differences between the regions of *PmF*-*box* and *PmSFB*. Firstly, two insertions of either five or two amino acid residues exist in the regions of *PmF*-*box* genes, that correspond to the *PmSFB* variable V1 and V2 domains, respectively. Secondly, a deletion of three amino acid residues was found in the putative region of *PmF*-*box* genes, corresponding to HVb in *PmSFB* genes. Lastly, the HVa domain in *PmF*-*box* genes had a deletion of one amino acid when compared with *PmSFB* genes (Fig. 4b). RT-PCRs for *PmF*-*box* genes were performed with cDNAs from pollen grains, leaves and styles of ‘Zaohong’, using the specific primers PmF1 and PmF2 (Fig. 1c). The results from these PCRs showed that *PmF*-*box* genes are expressed not only in pollen, but also in leaves and styles.

### Phylogenetic analysis

**Fig. 5:**
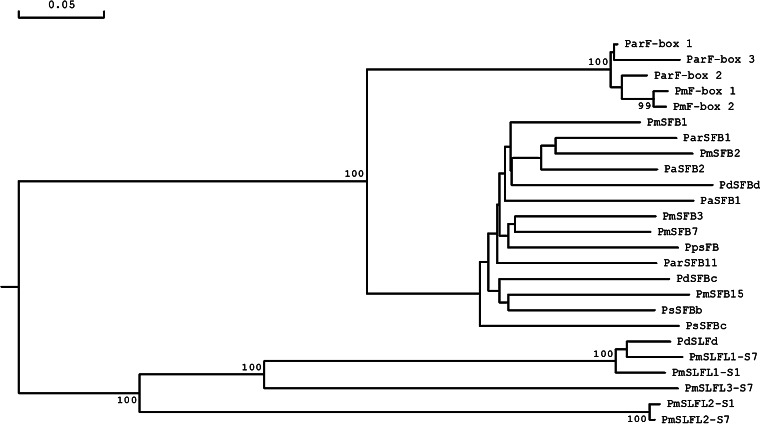
Phylogenetic tree of 25 *SFB/SLF(L)* and *F*-*box* genes in GSI plants based on aligned amino acid sequences using neighbor-joining. The *scale bar* represents 0.05 substitutions per amino acid site. The *numbers* at each branch indicate the percentage of 1,000 bootstrap replicates supporting the grouping at that node. GenBank accession numbers: *PaSFB*_*1*_ (AY805048), *PaSFB*_*2*_ (AY805049), *ParSFB*_*1*_(AY587563), *ParSFB*_*11*_ (EU652884), *PdSFB*_*c*_ (AB079776), *PdSFB*_*d*_ (AB081648), *PpsFB* (HM347516), *PsSFB*_*b*_ (AB252412), *PsSFB*_*c*_ (AB280792), *PmSFB*_*1*_ (AB101440), *PmSFB*_*3*_ (AB376968), *PmSFB*_*7*_ (AB101441), *PmSLFL1*-*S1* (AB092623), *PmSLFL1*-*S7* (AB092624), *PmSLFL2*-*S1* (AB092625), *PmSLFL2*-*S7* (AB092626), *PmSLFL3*-*S7* (AB092627), *PdSLF*_*d*_ (AB101660), *ParF*-*box1* (DQ422943), *ParF*-*box2* (DQ422944) and *ParF*-*box3* (DQ422945), the accession numbers of *PmSFB*_*2*_, *PmSFB*_*15*_ and two *PmF*-*box* genes were detailed in the text

A rooted phylogenetic tree of 25 genes including *SFB/SLF*(*L*) and *F*-*box* genes in GSI plants was constructed using aligned amino acid sequences by the neighbour-joining method (Fig. 5). Two major classes, the *SFB* clade and the *SLF*(*L*) clade, were found in the phylogenetic tree which demonstrate that *SFB/SLF*(*L*) and *F*-*box* genes share a common ancestor in *Prunus*. The tree also suggests that pollen determinant *SLF*(*L*) genes likely diverged earlier in *S*-*RNase*-based SI evolution.

*PmF*-*box* genes were clustered together with *ParF*-*box* genes and away from the *SLF/SLFL* genes, and appeared to be a sister group to the other *Prunus SFB* gene clade (Fig. 5). These findings indicate that *PmF*-*box* genes are not *SFB*-like genes but are instead a novel type of F-box gene with a higher sequence similarity to *SFB* than to *SLF/SLFL*.

## Discussion

### *PmF*-*box*, a novel F-box gene with sequence similarity to *SFB* alleles

A novel type of F-box gene (*PmF*-*box1* and *PmF*-*box2*) with sequence similarity to *SFB* was identified in ‘Zaohong’. The amino acid identities between *PmF*-*box* and *PmSFB* genes ranged from 61.03 to 64.65 %, similar to those between *PaF*-*box* and *ParFB* described by Vilanova et al. [25]. A putative F-box motif, two variable regions (V1 and V2) and two hypervariable regions (HVa and HVb) were identified in both *PmF*-*box* genes (Fig. 4b). The F-box motif, which contained many conserved residues, had previously been shown to be under purifying selection, and is essential for the formation of the SCF complex and subsequent protein degradation by the ubiquitin/26S proteasome pathway [41]. Meanwhile, V1, V2 and HVb of *SFB* have been shown to be hydrophilic and under positive selection, and might be the regions responsible for discrimination between self and non-self *S*-*RNases* [25, 42]. Thus, it seems that whatever is the role of PmF-box proteins, functions related to these putative domains could be changed or lost with regard to SFB proteins. In comparison to *PmSFB* genes, insertions and deletions were found in the putative regions V1 and V2, and HVa and HVb of *PmF*-*box* genes, respectively. These altered domains in *PmF*-*box* genes correspond to regions which are involved by *SFB* proteins in the specific recognition of the style counterpart, which might result in PPM in SC cultivars. Moreover, phylogenetic analysis suggests that this new type of F-box gene might play a novel, important role in the mechanism of GSI.

In summary, *PmF*-*box* was identified as a novel type of F-box gene. Further research into these genes could provide a deeper understanding of self and non-self recognition mechanisms in GSI as well as in various aspects of plant growth and development at the molecular level.

### *S*-locus external modifier factors might be involved in SC of ‘Zaohong’

In most *Prunus* fruit trees, SC is attributed to loss of function of style or pollen *S* genes. SPMs and PPMs have been shown to be associated with deletions or insertions in *S* alleles. In this study, we confirmed SC of ‘Zaohong’ and SI of ‘Xiyeqing’, through self-pollination tests based on averaged data collected over a 3-year survey. Cross-pollination tests were also carried out to study SC in ‘Zaohong’ using ‘Xiyeqing’, the SI cultivar with same *S* haplotypes (*S*_*2*_ *S*_*15*_). ‘Zaohong’ × ‘Xiyeqing’ displayed cross-incompatibility, but ‘Xiyeqing’ × ‘Zaohong’ showed cross-compatibility. Thus, the growth of pollen tubes of ‘Xiyeqing’ with the *S*_*2*_ or *S*_*15*_ haplotype was arrested in styles of ‘Zaohong’. However, the growth of pollen tubes of ‘Zaohong’ was not arrested when growing in ‘Xiyeqing’ styles, showing that SC in ‘Zaohong’ occurred in pollen, not in styles.

Using SRc-F/PM-C5 and F-BOX5′A/Pm-Vb, *S*-haplotypes were successfully cloned. No mutations leading to SC of ‘Zaohong’ were found in *S*-haplotypes. To investigate the loss of function of pollen *S* genes, the specific expression of *SFB* genes was tested in ‘Zaohong’ and ‘Xiyeqing’. The conclusion was that *SFB* genes of ‘Zaohong’ have a similar expression in pollen to that of *SFB* genes of ‘Xiyeqing’. Taken together, these findings indicate that the loss of function of pollen *S* genes causes SC of ‘Zaohong’, which is unrelated to both the mutation of the coding sequence and to the transcriptional level.

In addition to SPM and PPM, SC was also found to be associated with mutations at a modifier locus that is unlinked to the *S*-locus in *Prunus*. Indeed, Wünsch and Hormaza [24] and Wünsch et al. [27] have reported that the pollen-*S* component of the SC sweet cherry ‘Cristobalina’ is affected by a factor unlinked to the *S*-locus. In apricot meanwhile, Vilanova et al. [25] have also described a modifier locus affecting the function of the pollen *S* factor in the cultivar ‘Canino’, while Wu et al. [28] similarly found that unlinked factors caused a loss in pollen *S* activity in the cultivar ‘Katy’. In almond, a modifier locus affecting the expression of the *S*-*RNase* gene might be the cause of the breakdown of SI [26].

In the Solanaceae, an alternative means to SC has been demonstrated, where duplications of an *S*-allele led to competitive interactions that ultimately resulted in PPMs [6, 43, 44]. In addition, *S*-heteroallelic pollen is the cause of self-compatibility in some *Petunia* tetraploids [45]. Japanese apricot is a diploid species [46] and, with the exception of the *PmF*-*box* genes, there are no duplications of either *S*-locus gene in ‘Zaohong’. Vilanova et al. [25] rejected the possibility that SC in apricot, ‘Canino’ was due to *ParFB SFB*-like genes acting as duplicated pollen-*S* genes. However, this mechanism might still be involved in the SC of *Prunus*, as expression of these genes in pollen (or tubes) is observed, as well as changes in putative domains that correspond to regions of *SFB* proteins involved in the specific recognition of the style counterpart. Therefore, it appears that the loss of function of pollen *S* genes of ‘Zaohong’ is not tightly linked to the *S*-locus. However, modifier factors outside the *S*-locus, including the *PmF*-*box* genes, might be associated with SC of ‘Zaohong’.

## Abbreviations

*Pm*: *Prunus mume*
*Par*: *Prunus armeniaca*
*Pa*: *Prunus avium*
*Pd*: *Prunus dulcis*
*Ps*: *Prunus salicina*
*Pps*: *Prunus pseudocerasus*
